# QTc and QTcd Measurements and Their Relationships with Left Ventricular Hypertrophy in Hemodialysis Patients

**DOI:** 10.5935/abc.20180096

**Published:** 2018-07

**Authors:** Sora Yasri, Viroj Wiwanitkit

**Affiliations:** 1 KMT Primary Care Center, Bangkok, Tailândia; 2 Hainan Medical University, Haikou, China

**Keywords:** Hypertrophy, Left Ventricular, Renal Dialysis, Electrocardiography

Editor,

We read the publication on "QTc and QTcd Measurements and Their Relationships with Left Ventricular Hypertrophy in Hemodialysis Patients", which is very interesting.^1^ Alonso et al.^1^ concluded that "We found that QTc interval, in contrast to QTcd, is a reproducible and reliable measure and had a weak but positive correlation with LVMi in HD patients." This report used an unmatched control group; hence, the selection bias can be expected. In fact, the hypertrophy might be expected in a hemodialysis patient who might have underlying metabolic syndrome and vascular disease.^2^ To test the reproducibility, the repeated analysis is needed and there is a need to assess the within-run and between-run precision. In the present report, one cannot conclude that the test has a good reproducibility.
