# Supplementary material for: QTc and QTcd Measurements and Their Relationships with Left Ventricular Hypertrophy in Hemodialysis Patients
Source: Arq Bras Cardiol. 2018 Jul;110(6):602. doi: 10.5935/abc.20180096 (PMC6023640; doi:10.5935/abc.20180096)
Supplement: Related texts

# Reply

**Authors:** Maria Angélica Gonçalves Alonso, Valentine de Almeida Costa de Castro Lima, Maria Angela Magalhães de Queiroz Carreira, Jocemir Ronaldo Lugon

Thank you for your comments regarding our paper.

Our report was a case-control study with hemodialysis patients recruited from a single dialysis center and a control group matched by gender and age without overt kidney disease. For evaluation of the reproducibility and reliability of QTc and QTcd measures, intra- and inter-observer correlation and concordance tests were performed employing Pearson´s correlation, Cohen's Kappa coefficient and Bland Altman diagram. Two observers (unaware of the results from each other) manually measured the QT interval and its dispersion in the same electrocardiographic tracing at two different times with a one-week interval between measurements. Most of the previous studies that considered the reproducibility of the QTc and QTcd measurements, used only one method of evaluation, especially the correlation coefficient test without contemplating concordance tests. We applied three different types of tests, with two different observers coming to a likely conclusion of good QTc reproducibility. In contrast, QTcd does not seem to be a reliable and reproducible measurement.

The present study carries some limitations such as the relatively small number of patients and the exclusion criteria. Further studies performed on larger patient populations are needed to determine the optimal time to measure these parameters (pre-dialysis, during dialysis, or after dialysis), as well as the standardization of cutoff points for these parameters, techniques of measurements and correction for heart rate.

**Sincerely,**

Maria Angélica Gonçalves Alonso

Valentine de Almeida Costa de Castro Lima

Maria Angela Magalhães de Queiroz Carreira

Jocemir Ronaldo Lugon
